# Supplementary material for: The interactome of intact mitochondria by cross-linking mass spectrometry provides evidence for coexisting respiratory supercomplexes
Source: Mol Cell Proteomics. 2017 Dec 8;17(2):216–32. doi: 10.1074/mcp.RA117.000470 (PMC5795388; doi:10.1074/mcp.RA117.000470)
Supplement: Supplemental Data [file supp_RA117.000470_133922_0_supp_23978_fzffwf.zip › spectra_annotation/mito_spectra_annotation/486-1-1-1-24-1.pdf]

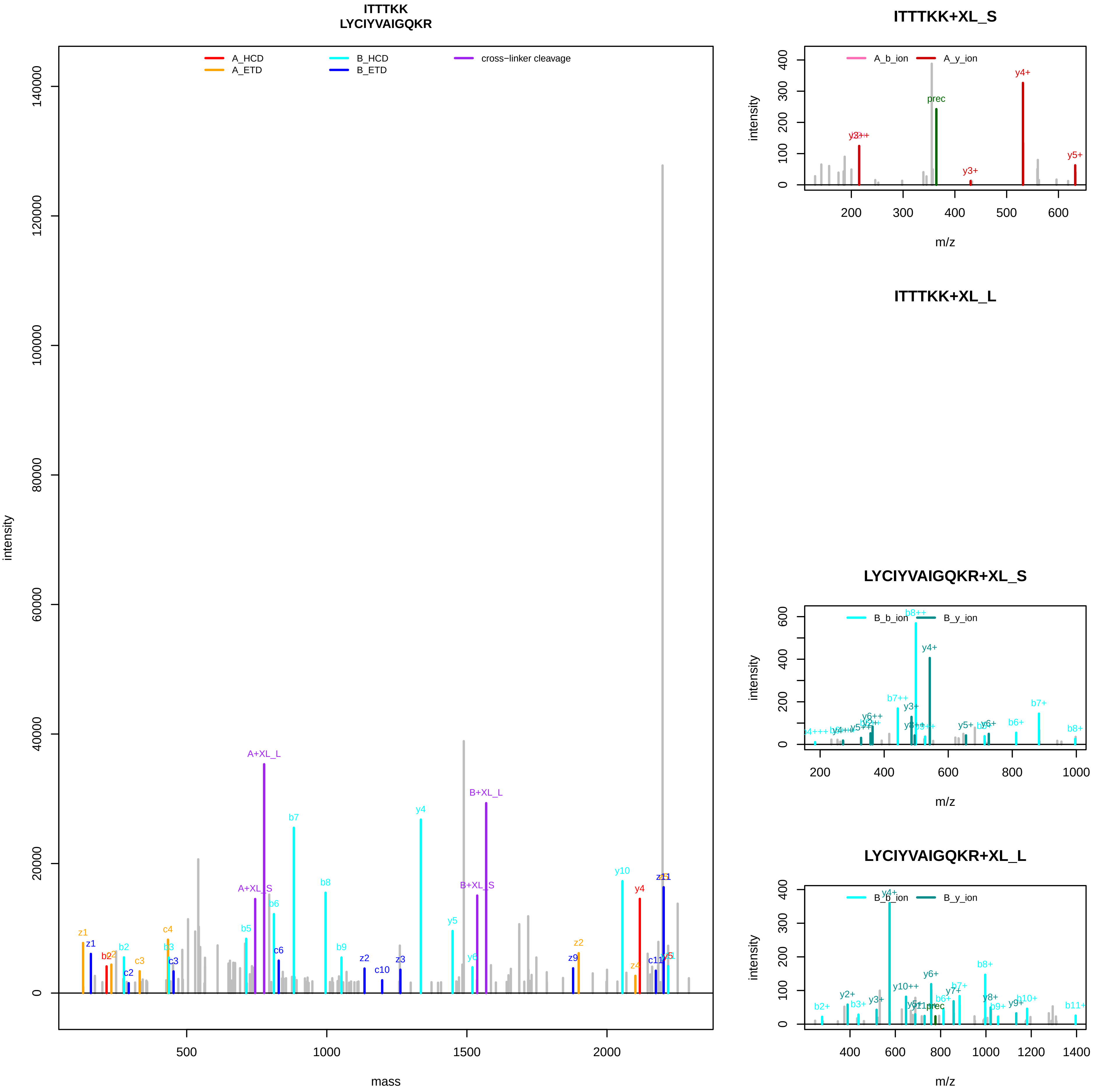

ITTTKK
LYCIYVAIGQKR
A_HCD
A_ETD
B_HCD
B_ETD
cross−linker cleavage
intensity
mass
0
20000
40000
60000
80000
100000
120000
140000
500
1000
1500
2000
z1
z1
b2
c2
b2
c2
c3
c4
b3
c3
b5
A+XL_S
A+XL_L
b6
c6
b7
b8
b9
z2
c10
z3
y4
y5
y6
B+XL_S
B+XL_L
z9
z2
y10
z4
y4
c11
z11
ITTTKK+XL_S
A_b_ion
A_y_ion
y4+
prec
y3++
y3+
y5+
m/z
0
100
200
300
400
200
300
400
500
600
ITTTKK+XL_L
LYCIYVAIGQKR+XL_S
B_b_ion
B_y_ion
b8++
y4+
b7++
y3+
y6++
y5++
y5+
y6+
b6+
b7+
b8+
0
200
400
600
200
400
600
800
1000
LYCIYVAIGQKR+XL_L
y4+
y2+
b2+
b3+
y3+
y10++
y6+
b7+
prec
b6+
y7+
b8+
y8+
b9+
y9+
b10+
b11+
0
100
200
300
400
400
600
800
1000
1200
1400
